# Mitochondrial-nuclear epistasis contributes to phenotypic variation in wild yeasts

**DOI:** 10.1186/1755-8166-7-S1-P40

**Published:** 2014-01-21

**Authors:** Swati Paliwal, Anthony C Fiumera, Heather L Fiumera

**Affiliations:** 1 Department of Biological Sciences, State University of New York at Binghamton, 4400 Vestal Pkwy E, Binghamton, NY 13902, USA

**Keywords:** Mitochondria, Phenotype, wild yeast

## Background

Mitochondria are ubiquitous organelles and are the main source of cellular energy. Mitochondria affect nearly every cellular process, including energy production, metabolite biosynthesis, ion homoeostasis, growth, cellular differentiation and apoptosis. In human and mice models, mitochondrial DNA variation is associated with various metabolic diseases. Mitochondrial functions require intricate interactions between mitochondrial (mt) and nuclear (n) genomes. The extent to which mitochondrial-nuclear (mt-n) interactions contribute to phenotypic variation in a population is unknown. We have made an attempt to find out if different combinations between the different mt and n genomes are found in natural populations would may lead to the expression of complex traits through epistatic interactions.

## Materials and methods

We created a novel population of 100 novel yeast strains with all possible combination of 10 naturally occurring and polymorphic, nuclear and mitochondrial genomes. These strains were phenotyped for metabolic growth under a variety of conditions, including high temperature, carbon source, and oxidative stress using high-throughput micro-cultivation plate readers. The results were analysed statistically for the growth rates using ANOVA.

## Results

It was found that mt-n epistasis significantly contributes to phenotypic differences among these strains, explaining up to 40% of phenotypic variation. A large number of strains were found to contribute, indicating the mt-n epistasis is a wide-spread phenomenon. The patterns of genome interactions vary across environments, indicating that multiple interactions affect fitness. Interestingly, we found that certain strains harboring their native mitochondrial genomes are more fit than when harboring non-native mtDNA, suggesting nuclear-mitochondrial co-evolution.

## Conclusions

It is suggested that mt-n epistasis may provide a fitness landscape upon which selection can operate. This work highlights the need to consider mt-n epistasis when characterizing the genetic basis behind complex traits and “missing heritability” and in developing treatments for mitochondrial disorders in humans.

